# Face-to-Face and Distance Education Modalities in the Training of Healthcare Professionals: A Quasi-Experimental Study

**DOI:** 10.3389/fpsyg.2018.01557

**Published:** 2018-08-22

**Authors:** Carmem L. E. Souza, Luciana B. Mattos, Airton T. Stein, Pedro Rosário, Cleidilene R. Magalhães

**Affiliations:** ^1^Health Sciences Program, Universidade Federal de Ciências da Saúde de Porto Alegre, Porto Alegre, Brazil; ^2^Public Health Department, Universidade Federal de Ciências da Saúde de Porto Alegre, Porto Alegre, Brazil; ^3^Department of Applied Psychology, School of Psychology, University of Minho, Braga, Portugal; ^4^Department of Education and Humanities, Universidade Federal de Ciências da Saúde de Porto Alegre, Porto Alegre, Brazil

**Keywords:** training of healthcare professionals, postgraduate, effectiveness of training course, distance education, face-to-face

## Abstract

This study evaluates the effectiveness of an 18-month-long course in Family Health. The course was offered in two modalities, distance education and face-to-face learning. Dependent variables were as follows: self-regulation of learning, procrastination, the perception of self-efficacy, and academic performance. The course was attended by 27 health professionals (i.e., physicians, nurses, and dentists) working in the Brazilian Unified Health System. The investigation followed a quasi-experimental design. Participants in the two modalities achieved similar academic performance; and globally no statistically significant differences were found regarding the study variables. Findings, notwithstanding their importance for professional training in health, are preliminary and further research is needed on the effectiveness of training modalities distance education and face-to-face learning (e.g., focus groups, interviews, online monitoring). The educational implications of this study are discussed and analyzed considering specificities and differences of each modality.

## Introduction

The Unified Health System was implemented in Brazil in 1990. The governmental law (i.e., No. 8080) established as major principles the universality of access to health care, the comprehensive care, and the equity of actions for all citizens (Brasil, 1990). To ensure that these principles were translated into practice several changes were triggered in the Public Health services. The main challenge of the Primary Health Care model is to promote the orientation of health practices and actions in a comprehensive and continuous way. These orientations are likely to improve the quality of life of Brazilian people (Lopes et al., 2007; Brasil, 2008, 2009, 2010, 2011).

The National Policy of Permanent Education in Health (NPPEH) (Brasil, 2004) has been working with the Unified Health System since 2004 to train health professionals in order to improve their practice. The NPPEH training model is consistent with the proposition that learning should not be separated from practice. For example, the NPPEH has been developing methodologies based on the working reality of health professionals to build connections between the learning processes and everyday practice (Conill, 2008; Lima et al., 2010; Batista and Gonçalves, 2011; Cardoso, 2012).

Based on the proposal for the Permanent Education policy; the Brazilian Ministry of Health has been stressing the need to reflect on the quality of the individual and collective care being delivered by the health system. Current research stresses that the care delivered needs to engage the whole community in the promotion and development of both educational and health practices (Ceccim, 2005a,b; Haddad et al., 2008; Cardoso, 2012; Miccas and Batista, 2014). Such assumptions are consistent with a social cognitive framework which considers individuals as agents in their health care, and committed to their own learning and development of autonomy (Bandura et al., 2008).

The study of self-regulated learning started in the 1980s with studies grounded in the evidence that cognitive capacities and skills did not sufficiently explain students’ performance (Zimmerman, 2002). Researchers were interested in examining in detail the role of the motivational dimensions of behavior and the self-regulation processes in students learning and academic success (Zimmerman, 2002; Rosário et al., 2012b).

The self-regulated learning framework stresses the agency of the individuals to attain their goals. Among other personal factors that help explain different levels of commitment with goals, this framework highlights individuals’ self-beliefs, which allows them to exercise some degree of control over their feelings, thoughts, and actions. Therefore, individuals are expected to display an agent role in this process being responsive to the environment and self-regulating behavior to reach self-set goals (Zimmerman, 1990; Bandura et al., 2008; Menendez, 2010 Sampaio et al., 2012).

Self-regulation is an active process in which individuals set goals that guide their learning to monitor, regulate, and control their cognition, motivation, and behavior to attain their goals (Rosário et al., 2012c). For these reasons, the training of healthcare professionals, when aligned with the principles of the Permanent Education Policy, should consider the self-regulation process.

When focusing on the training of health professionals, there are several elements that may interfere in the learning process. Procrastination, particularly, can be seen as a dysfunction of the self-regulation process. Some students procrastinate in their learning tasks, delaying or not completing their activities due, for example, to poor prioritization of tasks. This learning problem is likely to be associated with low outcomes (Sampaio et al., 2012).

Procrastination has a direct impact on students’ self-regulation process, and the latter influences the perception of self-efficacy (Bandura et al., 2008). In fact, self-efficacy influences behaviors, thoughts, and actions on a personal or professional level, also influencing self-regulation. A negative perception alienate the subjects from their goals either by not achieving the desired result or by abandoning the task. Whenever positive, self-efficacy guides actions to achieve goals even in the presence of obstacles.

The present study addresses an ongoing discussion in Brazil, and in other developing countries, regarding the need to increase the offer of online professional courses in health, as well as their efficacy, to respond to emerging training needs over a vast geographical area.

A few studies in Brazil have analyzed the impact of the modality of the delivery of training (distance or face-to-face) on the same course (Costa et al., 2014) or discipline (Nascimento et al., 2013); findings indicate no differences regarding performance while stressing specificities of each modality regarding student/content, student/teacher, and student/student dimensions.

The assessment of the outcomes of the face-to-face and EAD modalities in Brazil is carried out by the Ministry of Education. Data on prior evaluations regarding the results of the National Student Assessment show a leveling between the EAD and face-to-face modalities (Figueiredo, 2013). These results were consistent in subsequent years. However, in these studies participants were not assigned randomly, and findings did not address the quality of the processes displayed by students in these two modalities. Further investigation is needed to address variables that helps understand the leaning processes related to these two training modalities.

The present study analyzes the differences in the learning process and the learning outcomes of the two groups (distance and face to face). The learning outcomes were compared at three points in time (baseline, middle, and end of course) using declarative knowledge measures. The learning processes were compared in two moments (middle and end of the course) using three motivational variables (procrastination, self-regulation, self-efficacy).

## Materials and Methods

This study assessed the effectiveness of a training course offered to health professionals (physicians, nurses, and dentists) working in Primary Health care. The course was taught in two modalities [distance education (DE) and face-to-face (FTF)].

Our study was designed to examine the outcomes of the participants enrolled in the two modalities. Each of these modalities has particularities (i.e., relationships between students in the class) that may display distinct learning processes. Thus, to address the effectiveness of the modality of training delivery, data regarding declarative knowledge on the topics of the course, and a set of motivational variables was assessed. Prior research found no differences in the outcomes of the contents delivered through distinct training modalities, however, prior research did not assign participants randomly. In fact, self-assignment to a learning condition could help explain previous findings. Moreover, prior data is limited regarding motivational variables, thus there is no ground to set hypothesis. For these reasons, the current research should be considered a preliminary study.

Participants were required to have academic training in medicine, nursing, or dentistry and to be currently working in units of primary health care. No exclusion criteria were put in place. The course was free to primary health care workers, but participants could not opt for the modality of the course. After validation of the inscriptions, the participants were randomly assigned to a modality.

Sequences of study actions are as follows:

(1) Course disclosure by the State Department of Health
(2) Approved registrations: verification of records according academic training in medicine, nursing, or dentistry and to be currently working in units of primary health care
(3) Randomization of groups
(4) Baseline knowledge exam focused on the contents of the course
(5) Application of the instruments to analyze self-regulated learning, procrastination, self-efficacy, and academic performance in the middle and at the end of the course
(6) Completion of the course

The exam for the baseline knowledge of the participants was focused on their declarative knowledge on the topic Family Health. This exam was conducted prior to the beginning of the course; in fact, at this stage, candidates were not yet enrolled in the course. For this reason, the questionnaires to assess the motivational variables were applied in the middle and in the end of the course at the same time as the declarative knowledge tests.

Moreover, to stress the process nature and guarantee that the protocol for the assessment was followed by all participants in the same conditions, the motivational questionnaires were filled in by participants along with the declarative knowledge tests in the middle and in the end of the course. In fact, because our focus was on the process, the motivational variables were only assessed in the middle and end of course. It would be meaningless to measure them prior to the class starting. During the duration of the course, these were the two single moments when all participants were together in a room.

The effectiveness of the specialization course (Family Health) was analyzed using the following variables: self-regulation of learning, procrastination, self-efficacy, and academic performance, following a quasi-experimental model.

### Course Characterization

The number of qualified health professionals in Brazil specialized in Family Health is insufficient for the needs of the country. To close this gap and expand the possibilities of health professional training, a University in Southern Brazil developed a course in Family Health, using a distance learning modality sponsored by the Open University of the Unified Health System (Brasil, 2010). In a partnership with the Institute of Research and Education, and for the purposes of this study, the same course was offered to a comparison group in a face-to-face modality.

In both modalities, the contents, the pedagogical design, and the instructors were the same. The teaching methodology was based on case studies analysis. Both groups studied the same clinical cases.

Both courses ran for 18 months with a workload of 390 h. For the face-to-face group, all lectures were delivered by teachers in the classroom. The course for the distance education group was delivered on a website, accompanied by a tutor, and had three face-to-face meetings throughout the 18 months, two of which were for the assessment of the course.

### Participants

One hundred and eight candidates applied for the course on Family Health without prior notice of the learning modality they would undergo. Finally, 106 students enrolled in the course.

These participants were randomly assigned into the two groups by drawing lots; 53 participants were allocated to the group of the distance education modality; and 53 to the face-to-face modality. Of these, 64 decided to enroll, 38 in the distance education (DE) group and 26 in the face-to-face learning (FTF) group. Students who did not enroll explained that they quit because they were not allocated to the training modality of their choice.

Twenty-seven participants out of 64 (42%) successfully completed the course: 12 in the DE group and 15 in the FTF group. Student withdrawals were related to personal (not due to the learning modality) and professional reasons (e.g., work displacements).

Data collection was carried out by research assistants for both groups. The 15 students in the FTF group and 12 students in the DE group participated in all data collection stages.

The participants (88% women) were nurses (65%), dentists (23%), and physicians (12%). Moreover, 70% of the participants reported working in Family Health Strategy teams and 24% in traditional Basic Health Units. The average time of experience in Primary Health Care of these professionals ranged from 2 months to 3 years. The average work time in the current Health Team was 1 year and 9 months. Regarding the type of professional contract, 54% were public servants on the health care system, while the remaining 46% hold a precarious contract with the public health service.

**FIGURE 1:**
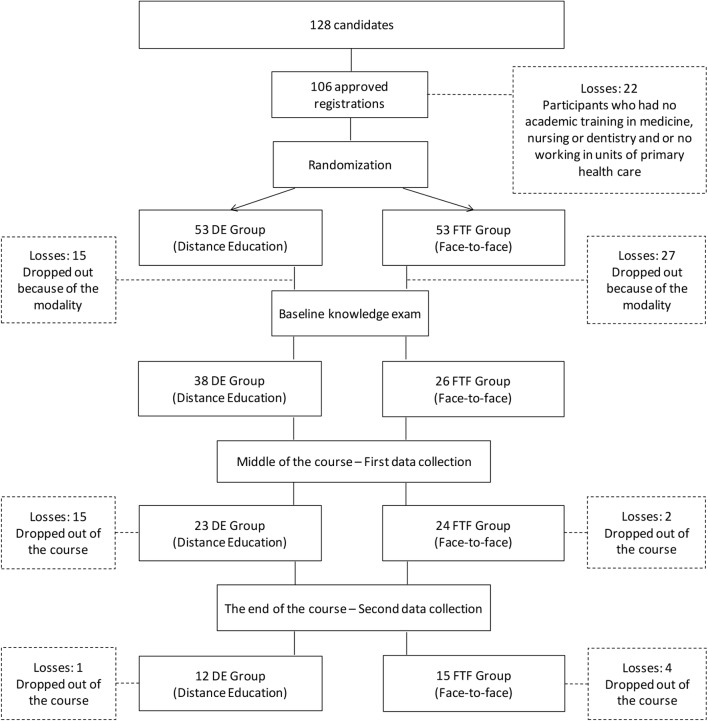
Flow diagram of the participants in the research stages (elaboration by authors).

### Instruments

The students reported use of self-regulated learning strategies was assessed with the Inventory of self-regulation (Núñez et al., 2011; Rosário et al., 2014). This questionnaire originally developed in Portugal (alpha = 0.87) is grounded on the cyclic model by Zimmerman (2002) and is comprised of 12 items. This questionnaire was validated to the population of Brazil (alpha = 0.75) (Sampaio et al., 2012). Students answered on a Likert scale from 1 (never) to 5 (always). A higher score indicated a higher use of self-regulated strategies. The items in the survey are related to the three phases of the cyclic process of self-regulation according to Zimmerman (2002), namely the Forethought Phase (e.g., “I make a plan before I start a report/task. I think about what I am going to do and what I need to get it done.”); Performance Volitional Control Phase (e.g., “In my study, I try to understand the material, make notes, summaries, solve questions/exercises that come in books/syllabi; ask questions about the material.”); and Self-Reflection Phase (e.g., “When I receive a grade, I think about concrete things that I have to do to improve my productivity/average.”). The internal consistency of the instrument for the present study was 0.75. The scores obtained for each of the dimensions correspond to the sum of their items.

The Academic Procrastination Scale originally developed in Portugal (Rosário et al., 2009) (alpha = 0.79) assesses students academic procrastination behaviors and was adapted for a Brazilian population by Sampaio et al. (2012). The questionnaire comprises 10 items distributed in two dimensions of five items each: Dimension I: Procrastination in daily study, deals with the postponement of execution of tasks in the classroom, school work, and continuous study. Dimension II: Procrastination in study before exams refers to behaviors which are likely to interfere with the study for the exam. Participants responded in a Likert scale of five points indicating the frequency of behavior between never (1) and always (5). The scores obtained for each of the dimensions correspond to the sum of their items. The interpretation of the scores shows that high levels indicate higher academic procrastination. The internal consistency of the instrument for the present study was 0.76.

The Self-efficacy scale was developed in Brazil (Sampaio et al., 2012) (alpha = 0.90) is a self-administered scale comprises 34 items to assess the perception of students regarding their capacity to cope with various aspects of the academic experience. The instrument has five dimensions: (A) academic self-efficacy; (B) self-efficacy in regulating learning; (C) self-efficacy in social interaction; (D) self-efficacy in proactive actions; (E) self-efficacy in academic management. Participants responded on a Likert scale of 10 points, in which 1 is barely able and 10 is very capable. The scores for each dimension are obtained by the sum of the responses for each of the items. The internal consistency of the instrument for the present study was 0.85.

The above instruments were applied in the middle and in the end of the course at the same time as the declarative knowledge used to assess students’ academic performance.

Academic performance was assessed as follows:

Baseline knowledge about Family Health content was assessed through a test with true/false and also direct questions, scored on a scale of 1 to 10. This test was developed and corrected by course teachers and was carried out before the beginning of the course.

Declarative knowledge tests (middle and end of the course) were developed and corrected by the course teachers. These tests were scored on a scale of 1 to 10 at the end of each module for the two topics covered: (I) Public Health (180 h/6 months into the class) and (II) Professional Training: clinical cases (180 h/12 months into the class). Moreover, students did a report on the topics covered during the training. This report was evaluated on a scale of 1 to 10 by a committee composed of two faculty. Each read and assessed the reports independently and met to reach a final consensus on the grade.

Normality tests and graphical analysis via Q–Q Plots showed that the data did not present normal distribution therefore non-parametric tests were used. The Mann–Whitney U test was used to compare the averages of the groups, the Wilcoxon U test was used for linkage analysis between groups at different times of the course and the Spearman Correlation (r) was also used. Data were analyzed with SPSS (Statistical Package for the Social Sciences) software for Windows (version 18.0).

### Ethics Statement

The study was evaluated and approved by the Federal University of Health Sciences of Porto Alegre Ethics and Research Committee (Registration 919/12), with consent for the course to be offered in the two modalities.

All participants gave written informed consent in accordance with the Declaration of Helsinki. There was no refusal to participate in the study at any time and the loss of study subjects occurred only as a result of students leaving the course.

## Results

### Preliminary Analysis

Results of the Self-Regulated Learning, Academic Procrastination, Self-efficacy, and Academic Performance variables are presented in **Supplementary Table S1**. The analyses showed no statistically significant differences between the variables in both groups, except in the following aspects:

(1) The procrastination dimension regarding studying for exams, in the middle of the course (*p* = 0.010);
(2) The total of the procrastination dimension, in the end of the course (*p* = 0.025);
(3) The perception of self-efficacy in social interaction, in the two moments (middle of the course *p* = 0.050; end of the course *p* = 0.037); and,
(4) The learning assessment of Module II, a course consisting of 180 h, which addressed knowledge concerning the *Professional training: clinical cases* in the second part of the course (*p* = 0.001).

The students’ self-regulated learning showed no significant difference between groups from the middle to the end of the course. However, this variable showed a negative correlation with the procrastination variable (DE group *r* = -0.65; FTF group *r* = -0.36) and a positive correlation with the knowledge of course content (DE group *r* = 0.33; FTF group *r* = 0.42).

The self-efficacy regarding the dimension concerning social interaction showed an increase (although not statistically significant) in the FTF group (**Supplementary Table S1**), (from *M* = 9.11; *SD* = 0.87 to *M* = 9.21; *SD* = 0.86). Such increase was not observed in the DE group (*M* = 8.33; *SD* = 1.18; *M* = 8.38; *SD* = 1.06). Regarding the assessment of Module II, the DE group had grade a point average of 8.98 (*SD* = 0.87), while the FTF group had a grade point average of 7.47 (*SD* = 0.67) on a 0 to 10 scale. The DE group showed increasing grade averages throughout the course, with grades ranging from 7.67 to 8.83. The FTF group average grade in Module I was 7.86, which decreased to 7.47 in the Module II. By the end of the course, the average assessment of the final papers was 8.77.

Considering the final course evaluation in relation to the prior knowledge, students in both groups showed increased knowledge of the *Family Health subject* (DE group *M* = 6.04, *SD* = 0.99 to *M* = 8.83, *SD* = 0.86; FTF group *M* = 6.23, *SD* = 0.90 to *M* = 8,77, *SD* = 0.70), still these differences were not statistically significant.

## Discussion

This study examined the effectiveness of a specialization course on Family Health delivered in two modalities (face-to-face and distance education). The effectiveness of the course in the two modalities was assessed through data regarding the declarative knowledge of the contents taught, and motivational variables (i.e., self-regulated learning, procrastination, and self-efficacy). The research question sought to understand to what extent the participation in different modalities could lead to different results, not only in content knowledge, but also in relation with the students’ motivation to learn.

In the current study, students in both groups completed the course with similar academic performance. In fact, the final academic results were similar despite the learning modality. Students in both modalities began the course with similar content knowledge, as identified by an assessment of baseline knowledge, and completed the course with similar results. Data indicates that students in both modalities attained the course’s learning objectives irrespective of the modality of the training. This finding is consistent with Cook’s studies (Cook, 2014) reporting that the effectiveness of a course is not simply determined by the modality or quality of its components, but by the context, objectives, and relevance of the training to participants. Moreover, a vast corpus of literature reports no differences in the students’ academic performance concerning the modality used to deliver content: distance education or face-to-face modes (Casagrande, 2008; Rezende and Dias, 2010; Nascimento et al., 2013; Costa et al., 2014; Kemp and Grieve, 2014; Kwant et al., 2015; Salajegheh et al., 2016; Shendell et al., 2016).

However, regarding the motivational processes, current data for both courses was not similar.

In the middle of the course, the FTF group scored higher than their counterparts on the self-regulated learning scale and lower on the procrastination scale, with a statistical significant difference in the procrastination dimension regarding the study for exams. Moreover, the correlation between self-regulation and procrastination was found to be negative. Findings on the self-regulated learning and procrastination variables indicate an inverse relationship between them.

The DE group presented intermediate results regarding the scores for these two scales, and their values were higher in the procrastination variable, which suggests that participants in the DE learning mode were less likely to use self-regulated strategies and more likely to procrastinate their study behavior. The correlation between the self-regulation and procrastination was negative still moderate, indicating that, whenever the student use of self-regulation strategies was lower, their procrastination behavior tended to increase. This finding is consistent with prior research on self-regulation of learning (Rosário et al., 2012c; Sampaio et al., 2012).

The students in the DE group enrolled for the first time in a distance education course, which may have influenced the results as they were expected to adapt their learning processes to a new learning modality and manage a set of learning strategies fit to this learning mode (Laguardia et al., 2010; Silva, 2010; Behar, 2013; Holanda et al., 2013). Participants were randomly assigned to this condition and no training on self-regulation addressing how to cope with an online learning mode was offered. Ad hoc evidence collected on informal discussions with students indicates that several participants struggled to cope with the challenges of this learning mode. These difficulties are consistent with data reported by Yang and Tsai (2010), which pointed to the complexity of the relationships between learning contents and learning strategies in a distance education mode. These authors investigated the impact of students’ conceptions of the outcomes of distance education in peer assessment, and found that more mature students with cohesive conceptions of their learning process are likely to make greater progress than students with fragmented views (Yang and Tsai, 2010).

In our study, the FTF group used and developed strategies in a familiar learning modality, while the DE group needed to learn concepts and use strategies in a totally new mode of delivering contents. Despite statistically non-significant, the FTF group showed a higher self-efficacy compared to the DE group.

Acknowledging that the DE group reported lower self-efficacy (in all dimensions) than their counterparts, a need for further studies of a qualitative nature is envisioned. Literature has shown that the beliefs and perception of self-efficacy can influence the learning process, either positively or negatively (Zimmerman, 1990; Bandura et al., 2008). Further studies examining students’ conception in relation to DE are expected to add to the literature.

Up to the middle of the course, academic performance was similar for both groups. In the second Module of the course, the DE group presented higher performance compared to the FTF group, although this difference was not statistically significant. Considering the adaptation to the modality experienced by the students in the DE group, and the procrastination reported, the latter finding is of relevance. This may be related to DE students self-regulation and self-efficacy (particularly in the dimension regarding proactive actions), which presented an increase in the final data collection, although no statistically significant differences regarding the first data collection were found.

In the end of the course, the FTF group maintained their reported use of self-regulated learning and decreased their procrastination (daily study). These students also reported to increase their academic self-efficacy. The DE students decreased their reported procrastination for daily study and study for exams, showing a tendency to manage time proactively, plan and use learning strategies; skills needed for undertake a distance education course with success (Palloff and Pratt, 2004; Rosário et al., 2009; Behar, 2013; Núñez et al., 2015).

Globally, we believe these results may have been influenced by the learning modality. Literature reports that learning challenges in the DE modality are twofold: learning specific content knowledge and learning how to study independently. This modality is fundamentally supported by written communication, which requires a good capacity for articulation and synthesis. Ideas are expected to be expressed with digital fluency, high autonomy, and parsimony. The lack of these skills can lead to withdrawal from the course. Such issues are not limited to the modality of distance education but are likely to become more evident due to their relevance to the development of the learning process in distance education (Laguardia et al., 2010; Silva, 2010; Behar, 2013; Holanda et al., 2013).

Extant research on self-regulation, and procrastination indicates that, in order to enhance these competencies, educational interventions focused on the construction of knowledge and on the agent role of the students are required. Current literature reports that to improve self-regulated learning students should be trained and encouraged to plan, execute, and assess their learning strategies (Rosário et al., 2012a, 2013).

The current study had important dropout rates in two distinct stages that are in need to be acknowledged: 60% of the candidates did not enroll in the course, and 53% of those who finally enrolled failed to complete the course. The numbers are relevant and should be addressed carefully. Several participants justified their non-enrolment due to the fact that there are not at ease with on-line training modalities. They were allocated to the online condition randomly, and their lack of familiarity with the online environments prevented their enrollment. Online courses allow institutions to extend training for populations that are geographically distant of the proponents of the training. Moreover, this training is cheaper to trainees, avoid displacements, and is likely to facilitate professional time management; however, as our data stress, prior to set online courses, it is important to consider the potential candidates perceived competencies to enroll in distance education, but also train them with self-regulated learning competences. This training is expected to help them cope with the challenges of this learning mode. In fact, approximately half of our participants did not complete the course. Reasons were of personal and professional nature. The sample had low experience on the topic (ranging from a few months to 3 years) which made them fit to the training, but also approximately half hold precarious professional contracts which help justify their early dropout from the training, these aspects may help explain findings.

Finally, while the DE modality carries new learning opportunities, it is still a study field that lacks further investigation. In medical education, research has already indicated that the use of technologies and mixed learning methods, or syllabi with complementary activities online, enhance learning (Boye et al., 2012; Heiman et al., 2012; Van Doorn and Van Doorn, 2014; Battat et al., 2016). Positive results using the online space for inter-professional education and collaborative cooperative learning were also observed by students (Solomon et al., 2010; Shendell et al., 2016; Wang et al., 2016). Still, further research is needed to deep our understanding on the reasons to enroll, the skills needed to cope with this learning mode, and the challenges faced by learners.

Another finding of relevance indicates that, against what was expected, both groups showed small differences in the reported self-efficacy regarding social interaction, in favor of the FTF group. Moreover, and surprisingly, within group differences for both groups were not found.

Due to the nature of the FTF modality, it was expected that the participants’ self-efficacy regarding social interaction was higher when compared with their counterparts in the DE group. Findings did not support this expectation. This is an important finding that alerts trainers and administrators to analyze not the nature of the learning mode delivered, but rather the activities conducted in the sessions (e.g., number and type of questions done in class, encouragement to students participation, on task feedback). For example, we found that participants (FTF and DE) worked and discussed the clinical cases in groups comprised by professionals with the same expertise (e.g., nurses with nurses, medical doctors with their colleagues). This methodology may have limited participants’ self-efficacy to interact with people with distinct health approaches to clinical cases. Future studies may wish to analyze the nature and organization of the activities run in the courses due to their potential influence in the learning process (see, Cook, 2014).

Globally, the findings of the current study are consistent with those by Menendez (2010), who analyzed the efficacy, efficiency, and effectiveness of a program in the face-to-face (CAPA) and distance education modalities (eCAPA). Menendez concluded that the virtual format, on a cognitive level, did not present a significant difference from the face-to-face format, but still promoted deep learning approaches and was more efficient to deliver content.

Despite preliminary, current data have important implications for institutions and individuals. The contribution of this paper to the field is in providing guidance around preparing healthcare professions for self-management in a DE learning environment (process). First, findings, show promising possibilities for continued education training irrespective to the learning mode. In fact, current data may help governments in developing countries, with vast territories such as Brazil, to run web platforms designed to deliver training to health professional. Brazil has a vast territory, and many health professionals lack opportunities to participate in face-to-face continued training due to the costs associated to displacement. This training mode could help diminish this problem; however, the training should include, besides contents focus on the professional development, intentional training on self-regulation learning strategies to help participants cope with the particular challenges of the DE. Current findings may also alert individuals willing to enroll in DE courses to the particular aspects of this learning mode; for example, to the need to display motivational resources and self-regulate learning strategies (e.g., volition and time management) to cope with the learning expectations.

With regard to the study limitations, the low number of participants and the important loss of participants during the course should be taken in consideration as was previously *mentioned*. These can be considered as attrition bias (Jüni and Egger, 2005) or as a result of the design of the study. In our study, participants did not choose the modality of the course and that may help explain the high dropout rate.

## Conclusion

In summary, the data presented are preliminary and require further attention from researchers; however, findings show a promising tendency that seems to support the option made by the Brazilian government to train professionals all around the country using the modality of distant learning. Future studies could consider running qualitative research to further investigate the phenomenon and promote the quality of the training for health professionals.

## Author Contributions

CS, LM, AS, PR, and CM developed the study design. CS and LM performed the data collection and follow-up analysis. CS wrote the manuscript with contributions from LM, AS, PR, and CM read and approved the final manuscript.

## Conflict of Interest Statement

The authors declare that the research was conducted in the absence of any commercial or financial relationships that could be construed as a potential conflict of interest.
