# Supplementary material for: Face-to-Face and Distance Education Modalities in the Training of Healthcare Professionals: A Quasi-Experimental Study
Source: Front Psychol. 2018 Aug 22;9:1557. doi: 10.3389/fpsyg.2018.01557 (PMC6113380; doi:10.3389/fpsyg.2018.01557)
Supplement: Supplementary file 1 [file Table_1.DOCX]

Supplementary Material

Face-to-face and distance education modalities in the training of healthcare professionals: a quasi-experimental study

Carmem L. E. Souza ^1*^, Luciana B. Mattos^1^, Airton T. Stein^2^, Pedro Rosário^3^, Cleidilene R. Magalhães^4^

^1^ Graduate in Health Sciences Program, Federal University Health Sciences of Porto Alegre (UFCSPA), Porto Alegre, Brazil

^2^Public Health Department, Federal University Health Sciences of Porto Alegre (UFCSPA), Porto Alegre, Brazil

^3^ Department of Applied Psychology, School of Psychology, University of Minho, Braga, Portugal

^4^ Education and Humanities Department, Federal University Health Sciences of Porto Alegre (UFCSPA), Porto Alegre, Brazil

*** Correspondence:**Carmem L. E. Souza
carmemlisiane@gmail.com

# Table 1

# Results of the dependent variables per/by group

| **Variables** | **DE Group** | | | **FTF Group** | | |  |
| --- | --- | --- | --- | --- | --- | --- | --- |
|  | **N** | ***M*** | ***SD*** | **N** | ***M*** | ***SD*** | ***p*** |
|  |  |  |  |  |  |  |  |
| **Motivational Variables** | | | | | | | |
| **Self-regulation of learning** |  |  |  |  |  |  |  |
| First data collection | 12 | 3.58 | 0.90 | 15 | 4.00 | 0.38 | .163 |
| Second data collection | 12 | 3.83 | .577 | 15 | 4.07 | 0.46 | .240 |
| **Procrastination - Dimension I (daily study)** | | |  |  |  |  |  |
| First data collection | 12 | 2.83 | 0.58 | 15 | 2.87 | 0.64 | .909 |
| Second data collection | 12 | 2.58 | .669 | 15 | 2.27 | 0.80 | .184 |
| **Procrastination - Dimension II (study for exams)** | | |  |  |  |  |  |
| First data collection | 12 | 3.17 | 0.58 | 15 | 2.53 | 0.52 | .010 |
| Second data collection | 12 | 2.92 | .515 | 15 | 2.60 | 0.51 | .131 |
| **Procrastination - Sum Total** |  |  |  |  |  |  |  |
| First data collection | 12 | 2.92 | 0.51 | 15 | 2.53 | 0.52 | .073 |
| Second data collection | 12 | 2.83 | .389 | 15 | 2.40 | 0.51 | .025 |
| **Perception of academic self-efficacy - Dimension A** | | | |  |  |  |  |
| First data collection | 12 | 7.99 | 1.13 | 15 | 8.37 | 0.78 | .366 |
| Second data collection | 12 | 8.20 | 0.97 | 15 | 8.81 | 0.68 | .070 |
| **Perception of self-efficacy in training regulation - Dimension B** | | | |  |  |  |  |
| First data collection | 12 | 8.33 | 1.00 | 15 | 8.62 | 0.88 | .525 |
| Second data collection | 12 | 8.50 | 0.97 | 15 | 8.83 | 0.86 | .390 |
| **Perception of self-efficacy in social interaction - Dimension C** | | | |  |  |  |  |
| First data collection | 12 | 8.33 | 1.18 | 15 | 9.11 | 0.87 | .050 |
| Second data collection | 12 | 8.38 | 1.06 | 15 | 9.21 | 0.86 | .037 |
| **Perception of self-efficacy in proactive actions - Dimension D** | | | |  |  |  |  |
| First data collection | 12 | 7.67 | 1.13 | 15 | 8.43 | 0.96 | .083 |
| Second data collection | 12 | 8.04 | 1.12 | 15 | 8.54 | 0.90 | .203 |
| **Perception of self-efficacy in academic management - Dimension E** | | | |  |  |  |  |
| First data collection | 12 | 8.00 | 1.21 | 15 | 8.80 | 0.94 | .064 |
| Second data collection | 12 | 7.83 | 1.03 | 15 | 8.67 | 1.23 | .053 |
| **Perception of self-efficacy- Sum total** | |  |  |  |  |  |  |
| First data collection | 12 | 8.02 | 1.03 | 15 | 8.62 | 0.75 | .136 |
| Second data collection | 12 | 8.22 | 0.85 | 15 | 8.81 | 0.74 | .124 |
| **Declarative Knowledge - Academic Performance** | | | | | | | |
| *Baseline knowledge* | 12 | 6.04 | 0.99 | 15 | 6.23 | 0.90 | .603 |
| *Knowledge of course content* |  |  |  |  |  |  |  |
| Module I: Field - Unit 1 (60h) | 12 | 7.67 | 0.73 | 15 | 7.86 | 0.83 | .844 |
| Module I: Field - Unit 2 (60h) | 12 | 8.03 | 0.79 | 15 | 7.76 | 0.77 | .267 |
| Module I: Field - Unit 3 (60h) | 12 | 8.02 | 0.66 | 15 | 7.95 | 0.75 | .750 |
| Module II: Professional Core (180h) | 12 | 8.98 | 0.87 | 15 | 7.47 | 0.67 | .001 |
| Final paper | 12 | 8.83 | 0.86 | 15 | 8.77 | 0.70 | .769 |

*N*: number; *M*: means; *SD*: Standard Deviations; *p*: Sig. (Wilcoxon)
